# Impact of the Plastein Reaction of Casein Hydrolysates in the Presence of Exogenous Amino Acids on Their Anti-Inflammatory Effect in the Lipopolysaccharide-Stimulated Macrophages

**DOI:** 10.3390/foods11020196

**Published:** 2022-01-12

**Authors:** Yun-Jiao Shi, Xin-Huai Zhao

**Affiliations:** 1 Key Laboratory of Dairy Science, Ministry of Education, Northeast Agricultural University, Harbin 150030, China; yunjiaoshi009@163.com; 2 School of Biology and Food Engineering, Guangdong University of Petrochemical Technology, Maoming 525000, China; 3 Research Centre of Food Nutrition and Human Healthcare, Guangdong University of Petrochemical Technology, Maoming 525000, China

**Keywords:** casein hydrolysates, plastein reaction, anti-inflammatory effect, macrophages

## Abstract

In this study, papain-generated casein hydrolysates (CH) with a degree of hydrolysis of 13.7% were subjected to a papain-mediated plastein reaction in the absence or presence of one of the exogenous amino acids—Gly, Pro, and Hyp—to prepare four plastein modifiers, or mixed with one of three amino acids to prepare three mixtures. The assay results confirmed that the reaction reduced free NH_2_ for the modifiers and caused amino acid incorporation and peptide condensation. When RAW264.7 macrophages were exposed to the CH, modifiers, and mixtures, these samples promoted macrophage growth and phagocytosis in a dose-dependent manner. In addition, the CH shared similar activity in the cells as the mixtures, while the modifiers (especially the PCH-Hyp prepared with Hyp addition) exerted higher potential than CH, the mixtures, and PCH (the modifier prepared without amino acid addition). The plastein reaction thus enhanced CH bioactivity in the cells. When RAW264.7 macrophages were stimulated with lipopolysaccharide (LPS), the inflammatory cells produced more lactate dehydrogenase (LDH) release and reactive oxygen species (ROS) formation, and caused more four inflammatory mediators (NO, PGE2, TNF-α, and IL-6) and two anti-inflammatory mediators (TGF-β1 and IL-10). However, the PCH-Hyp, PCH, and CH at dose levels of 100 μg/mL could combat against the LPS-induced inflammation. Overall, the PCH-Hyp was more active than the CH and PCH in reducing LDH release, ROS formation, and the secretion of these inflammatory mediators, or in increasing the secretion of the anti-inflammatory mediators. The qPCR and Western blot analysis results further confirmed that these samples had anti-inflammatory effects on the stimulated cells by suppressing the LPS-induced activation of the NF-κB signaling pathway, via regulating the mRNA/miRNA expression of iNOS, IL-6, TNF-α, IL-1β, COX-2, TLR4, IL-10, TGF-β1, miR-181a, miR-30d, miR-155, and miR-148, as well as the protein expression of MyD88, p-IKKα, p-IκBα, p-NF-κB p65, and iNOS, involved in this signaling pathway. In addition, the immunofluorescence assay results revealed that these samples could block the LPS-mediated nuclear translocation of the p65 protein and displayed the same function as the NF-κB inhibitor BAY 11-7082. It was concluded that CH could be endowed with higher anti-inflammatory activity to the macrophages by performing a plastein reaction, particularly that in the presence of exogenous Hyp.

## 1. Introduction

The plastein reaction was discovered by Danilevski and Okuneff in 1902, when the addition of rennet into the protein hydrolysate led to the production of a precipitate [1]. This reaction meant a process in which a high substrate concentration (20–50%, *w*/*v*) of protein hydrolysate or polypeptide was catalyzed by peptide bonding mode in the presence of an appropriate protease to form an insoluble, high molecular weight, protein-like precipitate, thixotropic colloids, or thixotropic viscous gels [2,3]. This reaction can modify the amino acid sequences and spatial structures of proteins or peptides, change the nutritional values of proteins or peptides by enforcing the restrictive amino acids, or remove the unwanted amino acids or other components from the specific products. The plastein products from protein hydrolysates has traditionally relied on the use of enzymes, such as neutrase, alkaline protease, papain, and bromelain [4]. Many protein-rich food ingredients have now been used as substrates for plastein reaction, such as casein, soybean, and pig blood [5,6,7], among others. This reaction thus affects several properties of proteins, including surface hydrophobicity, thermal stability, gelation, and metal chelating ability [3,4,5,6]. A review from Udenigwe and Rajendran has given an efficient background on this reaction and its potential applications [7]. This reaction can also cause bioactivity changes in protein hydrolysates. For example, it was observed that the plastein reaction of the chicken plasma protein and sea cucumber hydrolysates led to improved inhibition of the angiotensin-converting enzyme (ACE) [8,9], and that of soy protein hydrolysates resulted in higher anti-platelet activity [10]. Moreover, the plastein products enriched with exogenous phenylalanine (Phe) or tyrosine (Tyr) could provide a protective activity in the hepatocytes against the H_2_O_2_- or ethanol-induced damage [11,12]. In addition, this reaction was evident to elevate the anti-oxidative and hypolipidemic effects of protein hydrolysates [13,14]. Overall, this reaction has the potential to improve several bioactivities of protein hydrolysates or peptides efficiently. To further reveal the possible application of this unique chemical reaction on protein hydrolysates or peptides, it is necessary to deeply understand and investigate elements of the plastein reaction, such as the critical anti-inflammatory function of protein hydrolysates or peptides.

The process of inflammation in the body is a natural immune response to toxins, pathogens, allergens, and stimulated tissues [15]. An inflammatory response can activate the release of these cell-derived mediators such as tumor necrosis factor-α (TNF-α), interleukin-6 (IL-6), nitric oxide (NO), and prostaglandin E2 (PGE2) [16]. Meanwhile, it can also inhibit the release of these anti-inflammatory mediators, such as transforming growth factor β1 (TGF-β1) and IL-10 [16]. In general, NF-κB is a nuclear transcription factor discovered in the nuclear extracts of the B lymphocytes, which is regarded to regulate the inflammatory response known as a “central regulator of the immune response” [17]. The regulation of the NF-κB signaling pathway can thereby influence the inflammatory response. Several bioactive components were reported to mediate the expression of the targeted genes and related proteins in the NF-κB signaling pathway or mediate the expression of several inflammatory cytokines regulating the NF-κB signaling pathway or those suppressing excessive inflammatory responses [18,19,20]. For example, the hydrolysates from fish and rice proteins could inhibit NF-κB activation, reduce p65 translocation to the nucleus, block NF-κB signaling pathway, and exert anti-inflammatory function [21,22]. Therefore, the question of whether the plastein reaction (especially that occurs in the presence of exogenous amino acids) can change the anti-inflammatory effect of casein hydrolysates attracts our attention.

Lipopolysaccharide (LPS), as the most important endotoxin, is the major component of the outer membrane of Gram-negative bacteria. LPS can stimulate RAW264.7 macrophages to induce inflammation and is widely used to establish an inflammatory model [23,24]. In addition, glycine, proline, and hydroxyproline have been reported to have anti-inflammatory, immunomodulatory, and cytoprotective effects [23,24,25]. In this study, we prepared the casein hydrolysates using papain and then modified the hydrolysates in the presence and absence of one of three exogenous amino acids—glycine (Gly), proline (Pro), or hydroxy-proline (Hyp). Afterwards, in vitro anti-inflammatory activities of these products were assessed using LPS-stimulated RAW264.7 macrophages as cell model and unmodified casein hydrolysates as a control. These indices, such as growth proliferation, lactate dehydrogenase (LDH) release, reactive oxygen species (ROS) level, macrophage phagocytosis, and the secretion of NO, PGE2, TNF-α, IL-6, TGF-β1, and IL-10, were detected to reflect cell inflammation and anti-inflammatory activities of the targeted products, while the expression levels of MyD88, p-IκBα, p-NF-κB p65, p-IKKα, and iNOS, involved in the NF-κB signaling pathway, were detected. This study aimed to reveal whether the plastein reaction could impact the anti-inflammatory potential of protein hydrolysates efficiently and whether the three exogenous amino acids might cause different anti-inflammatory efficiencies.

## 2. Materials and Methods

### 2.1. Materials

Caseinate (protein content 955.0 g/kg, dry basis) was purchased from Beijing Aoboxing Biotechnologies Inc. (Beijing, China). The LPS, neutral red, and Dulbecco’s modified Eagle medium (DMEM) were acquired from Sigma-Aldrich Co. Ltd. (St. Louis, MO, USA). The fetal bovine serum (FBS) was purchased from Wisent Inc. (Montreal, QC, Canada), while the Gly, Pro, and Hyp (in the form of esters) were purchased from Aladdin-Reagent Co. Ltd. (Shanghai, China). The trypsin-EDTA and phosphate-buffered saline (PBS) were obtained from Solarbio Science and Technology Co. Ltd. (Beijing, China), and the papain (detected activity of 30 kU/g) was purchased from Sinopharm Chemical Reagent Co. Ltd. (Shanghai, China). All reagents were of analytical grade unless otherwise stated.

Cell counting kit-8 (CCK-8), Prime Script RT reagent kit and SYBR Premix Ex Taq (Tli RNaseH Plus) were purchased from Dojindo Molecular Technologies Inc. (Kyushu, Japan). The LDH detection kit was purchased from Nanjing Jiancheng Bioengineering Institute (Nanjing, China). The Ponceau S staining solution and kits used to assay protein content, ROS, and NO were obtained from Beyotime Institute of Biotechnology (Shanghai, China). The ELISA kits for cytokine and mediator detection were purchased from Wuhan Boster Biological Engineering Co. Ltd. (Wuhan, China). The RNAprep pure cell/bacteria kit was purchased from Tiangen Biotech Co. Ltd. (Beijing, China). The MiPure Cell/Tissue miRNA kit, miRNA 1st Strand cDNA Synthesis kit, and miRNA Universal SYBR qPCR Master Mix were purchased from Vazyme Biotech Co. Ltd. (Nanjing, China).

The primary antibodies (β-actin bs-0061R, MyD88 bs-1047R, phospho-NF-κB-p65 (Ser536) bs-0982R, and iNOS bs-0162R) were provided by Bioss Biotechnology Co. Ltd. (Beijing, China), while phospho-IκBα (Ser32) 2859T and phospho-IKKα/β (Ser176/180) 2694T) were obtained from Cell Signaling Technology Co. Ltd. (Danvers, MA). Goat anti-rabbit secondary antibody was purchased from Abcam PLC (Cambridge, UK), while NF-κB inhibitor (BAY11-7082) was purchased from Abmole Bioscience Co. Ltd. (Houston, TX). The DAPI, primary antibodies (NF-κB p65) and CY3 goat anti-rabbit secondary antibody were provided by Servicebio Bioscience Co. Ltd. (Wuhan, China).

### 2.2. Preparation and Plastein Reaction of Casein Hydrolysates

The caseinate was dispersed in water to achieve 50 g/L, adjusted to pH 7.0 by 0.5 mol/L HCl, added with papain of 3.5 kU/g protein, kept at a water bath of 60 °C for 8 h to perform caseinate hydrolysis, and then heated in a boiling water for 15 min to stop hydrolysis. After cooling, the hydrolysates were adjusted to pH 7.0 and centrifuged at 11,000× *g* for 20 min. The supernatant was freeze-dried and heat-dried at 105 °C to obtain the casein hydrolysates (CH), which were stored at −20 °C until needed.

The plastein reaction of CH was carried out in the presence or absence of exogenous Gly, Pro, and Hyp, under the same reaction conditions as a previous work [25]. In brief, CH concentration of 40% (*w*/*w*), 0.6:1 (moL/moL) ratio of the added amino acids to the amino groups of CH, and papain usage of 3.5 kU/g protein were used in the reaction. The reaction was performed at pH 7.0 and 37 °C for 6 h. After heating in a boiling water bath for 15 min, the whole reaction mixture was cooled, freeze-dried, and then heat-dried at 105 °C to obtain the products of the plastein reaction (i.e., the plastein modifiers). In this study, PCH represents the modifier in absence of exogenous amino acids, while PCH-Gly, PCH-Pro, and PCH-Hyp represent the modifiers in the presence of Gly, Pro, and Hyp, respectively. Moreover, the mixtures of CH, inactivated enzyme and one of Gly, Pro, and Hyp without the plastein reaction were also prepared and designed as CH-Gly, CH-Pro, and CH-Hyp, respectively.

### 2.3. Cell Culture and Treatment

The RAW264.7 macrophages, provided by the Cell Bank of the Chinese Academy of Sciences (Shanghai, China), were cultured at 37 °C with the complete DMEM medium containing 10% FBS in a 5% CO_2_ environment. The medium was routinely replaced every 1–2 days. The cells cultured only by the medium served as negative control, while those treated by 1 μg/mL LPS were considered as the positive model.

### 2.4. Assays of Protein and Free Amino Group Contents

Nitrogen content was determined using the Kjildahl method [26] and converted to protein content using a conversion factor of 6.38. Meanwhile, free amino group (−NH_2_) content (mmol/g protein) was measured using the o-phthaladehyde method, as previously described [27], at a UV-2401PC spectrophotometer (Shimadzu, Kyoto, Japan) at 340 nm. The degree of hydrolysis of CH was calculated as previously described [28].

### 2.5. Determination of Growth Proliferation and Macrophage Phagocytosis

The cells of 100 μL were inoculated in 96-well plates at a density of 5 × 10^4^ cells/well and cultured for 24 h. The assessed samples were dissolved in the medium to reach dose levels of 25–100 µg/mL and the cells were treated for 24 h. Afterwards, 10% CCK-8 solution of 100 μL (10 μL CCK-8 with 90 μL the medium) was added to each well and incubated for 1.5 h. The optical density values were measured at 450 nm by a microplate reader (Bio-Rad Laboratories, Hercules, CA, USA) and used to calculate cell viability. In this study, the cell viability of the control group was regarded as 100%.

The cells were inoculated into 96-well plates, as above, and incubated for 24 h. After discarding the supernatant, the cells were pretreated with the samples at 25–100 µg/mL for 30 min and then stimulated by 1 µg/mL LPS for 24 h. After discarding the supernatant, 100 µL of 1% neutral red solution (dissolved in physiological saline) was added to each well while the cells were incubated for 30 min followed by a PBS (0.01 mol/L, pH 7.2) wash, 3 times. Cell lysis solution (1% glacial acetic acid: anhydrous ethanol = 1:1, *v*/*v*) of 100 µL was added to incubate the cells for 2 h. The absorbance value was measured at 450 nm using the microplate reader. The phagocytosis index (PI) was determined as previously described [29].

### 2.6. Determination of LDH Release and ROS Level

The cells were inoculated into 96-well plates, incubated, and treated as above, but using a sample dose of 100 µg/mL. After centrifugation at 190× *g* for 5 min, the supernatants were collected to measure the LDH activity according to the instruction provided by the kit manufacturer. LDH release of the control cells was set as 100% [30].

The cells were inoculated into 6-well plates at a density of 1 × 10^6^ cells/well and incubated for 24 h. After the removal of the supernatants, the cells were treated with the samples at 100 µg/mL for 24 h and 1 µg/mL LPS for 24 h. After centrifugation at 190× *g* for 5 min, the cells were washed with PBS 3 times, incubated with 2′-,7′-dichlorodihydrofluorescein diacetate (5 µmol/L) for 20 min at 37 °C in the dark, and washed by the serum-free medium 3 times. A fluorescent microplate reader (Infinite 200, Tecan, Männedorf, Switzerland) was used to measure the fluorescence intensity at the excitation/emission wavelengths of 488/525 nm. The ROS level was expressed as previously described [31].

### 2.7. Measurement of NO, PGE2, TNF-α, IL-6, TGF-β1, and IL-10 Secretion

The cells inoculated into 6-well plates (1 × 10^6^ cells/well) were incubated for 24 h until adhered. After discarding the supernatants, the cells were treated with the samples at a dose level of 100 µg/mL for 24 h and 1 µg/mL LPS for 24 h. The cells were centrifuged at 2000× *g* for 20 min to collect supernatants, which were measured for NO, PGE2, TNF-α, IL-6, TGF-β1, and IL-10 using the respective ELISA kits and instructions.

### 2.8. Quantitative Real-Time PCR Analysis

The cells (2 mL, 1 × 10^6^ cells/well) were inoculated into 6-well plates and incubated for 24 h. After discarding the supernatants, the cells were treated with the samples of 100 μg/mL for 24 h and 1 µg/mL LPS for 24 h. The total RNA and miRNA were extracted using the RNAprep pure cell/bacteria kit and MiPure Cell/Tissue miRNA kits, respectively. The reverse transcription was conducted according to the instructions from the PrimeScipt RT reagent kit and miRNA 1st Strand cDNA Synthesis Kit to obtain cDNA, while the specific primers (Table 1) were used to amplify the cDNA. The RT-PCR analysis was conducted at the Applied Biosystems 7300 Real-Time PCR (ABI, Foster City, CA, USA), using SYBR Green Master Mix kit (Takara, Japan), miRNA Universal SYBR qPCR Master Mix kit, and respective protocols. The U6 and β-actin were used as two internal references, while relative expression level was calculated using the 2^−ΔΔCt^ method, as previously described [32].

### 2.9. Western Blot Analysis

The cells (10 mL, 5 × 10^5^ cells/well) were inoculated in 10 cm cell culture dishes, incubated for 24 h, treated by the samples of 100 μg/mL or NF-κB inhibitor (BAY11-7082, 2.5 μmol/L) for 24 h, and then exposed to 1 µg/mL LPS for 24 h. The cells were lysed with a pre-cooled RIPA cell lysate containing 1 mmol/L PMSF, and the supernatants were collected using a 14,000× *g* centrifugation for 5 min. Total protein content was measured using the BCA protein kit. The extracted proteins (40 μg) were separated using 12% SDS-PAGE gels and then transferred to nitrocellulose membranes. The target bands were placed in the Ponceau S stain solution for 2 min and sealed using 5% skim milk at 37 °C for 2 h. After washing with TBS/T (TBS with 0.1% Tween-20, 1×), the target bands were incubated with the primary antibodies (1:1000 dilution) at 4 °C overnight. After washing with TBS/T, the membranes were incubated for 1 h at 37 °C with the secondary antibody (1:10,000 dilution). Ultimately, the target bands were visualized using an Amersham Imager 600 (General Electric Company, Boston, MA, USA), while protein expression levels were standardized to β-actin.

### 2.10. Immunofluorescent Analysis

The cells (2 mL, 1 × 10^4^ cells/well) were inoculated on 6-well plates with coverslips (15 mm diameter, 0.13–0.16 mm thickness) and incubated for 24 h. After treatment with the samples of 100 μg/mL or NF-κB inhibitor (BAY11-7082, 2.5 μmol/L) for 24 h and 1 µg/mL LPS for 24 h, the cells were washed with the PBS and blocked with 3% BSA at 20 °C for 30 min. Then, the cells were incubated with the primary antibody p65 (1:200 dilution) overnight at 4 °C, the secondary antibody CY3 goat anti-rabbit (1:300 dilution) for 50 min, and then incubated with DAPI stain at 20 °C for 10 min. The images were then captured using a fluorescence microscope (Olympus, Tokyo, Japan).

### 2.11. Statistical Analysis

All experiments and assays were performed at least three times, while the data were expressed as means or means ± standard derivations. Significant differences of means from different groups were analyzed using the SPSS 16.0 software (SPSS Inc., Chicago, IL, USA), while one-way ANOVA and Duncan multiple test comparison were used to determine the significant difference (*p* < 0.05).

## 3. Results

### 3.1. Chemical Features of the Modifiers and Their Effects on Cell Growth and Phagocytosis

The −NH_2_ contents of these prepared samples were measured to exhibit their different chemical features (Table 2). The CH had a −NH_2_ content of 1.160 mmol/g protein, resulting in a degree of hydrolysis of 13.7%. However, after the performed plastein reaction, the resultant PCH had a −NH_2_ content of 0.935 mmol/g protein. The decreased −NH_2_ content (0.225 mmol/g protein) confirmed that the PCH had peptide condensation (one of the three reactions involved in the plastein reaction); therefore, the PCH reasonably had a lower −NH_2_ content. Meanwhile, a mixing of the CH with one of the three exogenous amino acids brought more −NH_2_ (from the used amino acids) for the three mixtures. As a result, the 3 mixtures had very close −NH_2_ contents of 1.792–1.820 mmol/g protein. Moreover, the plastein reaction of the CH and one of the exogenous amino acids endowed the 3 modifiers with −NH_2_ contents of 1.433–1.456 mmol/g protein. Clearly, the 3 modifiers had reduced −NH_2_ contents about 0.36 mmol/g protein, compared with the respective mixtures. Referring to the −NH_2_ content decrease in the PCH, it was concluded that the modifiers had the peptide condensation (−NH_2_ content decrease) and amino acid incorporation (further −NH_2_ content decrease) simultaneously. All results supported that the modifiers (PCH, PCH-Gly, PCH-Pro, and PCH-Hyp) had different chemical features from the CH and the three mixtures (CH-Gly, CH-Pro, and CH-Hyp), thereby, might possess changed activity in the cells.

To evaluate the possible effects of these assessed samples on macrophage growth, three dose levels (i.e., 25, 50, and 100 µg/mL) were used to treat the macrophages. The growth proliferation of these samples at each dose level on the cells is expressed as cell viability and given in Table 3. Overall, all samples at these dose levels had no cytotoxicity to the cells but could exhibit a dose-dependent promotion of macrophage growth. However, these samples had different abilities to promote cell growth. Compared with the corresponding mixture, the plastein modifiers promoted cell growth significantly at the same dose level. When the cells were exposed to the CH or PCH, the cells showed cell viability of 102.5–111.7% or 111.8–119.1%, respectively. The PCH showed higher activity than the CH to promote macrophage growth. Comparison of the data also demonstrated that the three modifiers, PCH-Gly, PCH-Pro, and PCH-Hyp, were more active than the PCH, because they led to cell viability of 114.3–124.4%, 113.7–122.9%, and 115.4–129.7%, respectively. However, the three mixtures (CH-Gly, CH-Pro, and CH-Hyp) consistently showed slightly higher activity than the CH in promoting macrophage growth, but lower activity than PCH. Thus, it can be concluded that it was the plastein reaction (especially in the presence of the exogenous amino acids) that conferred the higher activity of the modifier on macrophages, while Hyp usage could yield the highest activity for the resultant modifier PCH-Hyp. In addition, the dose level of 100 µg/mL always caused higher growth proliferation for the macrophages than other dose levels.

To evaluate the possible impacts of these samples on the phagocytosis function of the macrophages stimulated by LPS, the cells were exposed to them at 3 dose levels (25, 50, and 100 μg/mL) before the LPS treatment. The values of the phagocytotic index (PI) are given in Table 4. Compared with the control cells without LPS stimulation (PI value 1.00), model cells with LPS stimulation had promoted phagocytosis (PI value 2.15), reflecting the LPS-induced inflammation. However, all samples assessed showed the ability to inhibit the phagocytosis of the LPS-stimulated cells in a dose-dependent manner, suggesting an anti-inflammatory function of these samples. To be more specific, when the cells were exposed to the CH or PCH, the phagocytic index (PI) was reduced to 1.95–2.01 or 1.72–1.77. The PCH clearly exhibited stronger activity than the CH inhibiting the phagocytosis of the inflammatory cells. The results also demonstrated that the PCH-Gly, PCH-Pro, and PCH-Hyp had higher activity than the PCH, because they led to decreased PI values of 1.61–1.73, 1.70–1.75, and 1.52–1.71, respectively. In addition, the 3 mixtures (CH-Gly, CH-Pro, and CH-Hyp) showed lower anti-inflammatory activity than the PCH but higher anti-inflammatory activity than the CH, because they brought about PI values between 1.81 and 1.94. The performed plastein reaction (especially in the presence of the exogenous amino acids) thus endowed the modifiers with a higher inhibitory effect on the phagocytic activity of the stimulated macrophages, while the reaction with Hyp addition caused the highest inhibitory ability for the resultant modifier PCH-Hyp.

Based on these obtained results (Table 3 and Table 4), the dose level of 100 µg/mL was used in further anti-inflammatory evaluations to treat the macrophages, while 3 samples of CH, PCH, and PCH-Hyp were evaluated and compared for their anti-inflammatory potentials. The other two modifiers and the three mixtures were no longer evaluated in this study.

### 3.2. LDH Release and ROS Formation in Macrophages in Response to the LPS and Modifiers

**Figure 1:**
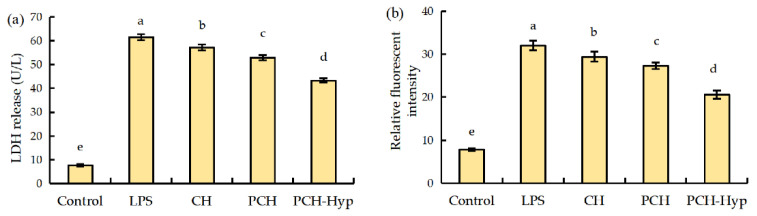
LDH release (**a**) and ROS production (**b**) in the macrophages exposed to LPS and the plastein modifiers. CH—casein hydrolysates; PCH—the modifier of CH; PCH-Hyp—the modifier of CH in the presence of Hyp. Different lowercase letters above columns indicate significant differences (*p* < 0.05).

To illustrate the other activity of these samples on the LPS-stimulated macrophages, the cells were exposed to 100 μg/mL of the CH, PCH, or PCH-Hyp before the LPS stimulation and then measured for their LDH release and ROS levels. The detected LDH release is given in Figure 1a. Compared with the control cells without LPS stimulation (7.5 U/L), the LPS-stimulated cells (i.e., model cells) had increased LDH release (61.5 U/L). However, the CH, PCH, and PCH-Hyp all could inhibit LDH release, resulting in decreased LDH release of 57.2, 52.9, and 43.3 U/L, respectively. Apparently, the PCH-Hyp and CH showed the highest and lowest activity in cells, respectively. Hence, the performed plastein reaction (especially with Hyp addition) is suggested endowing the modifiers with increased activity to combat against LPS stimulation and thereby reduce LDH release. Meanwhile, intracellular ROS levels of the cells in different groups were also measured (Figure 1b). As expected, LPS stimulation resulted in enhanced ROS levels (or oxidative stress) in the model cells as the relative fluorescence intensity value increased from 7.8 (control cells) to 32.0 (model cells). Using the CH, PCH, and PCH-Hyp at 100 μg/mL in the cells caused less ROS generation; subsequently, the detected ROS levels were reduced to 29.4, 27.3, and 20.6, respectively. The PCH-Hyp and CH led to the highest and lowest reduction in ROS in cells, implying the plastein reaction (especially with Hyp addition) enhanced the activity of the modifiers efficiently. All results consistently indicated that the plastein reaction (especially with Hyp addition) had an ability to enhance CH activity to the LPS-stimulated cells, including the ability to protect membrane integrity (reduced LDH release) and attenuate oxidative stress (reduced ROS formation).

### 3.3. Effect of the Modifiers on Inflammatory and Anti-Inflammatory Mediators in LPS-Injured Macrophages

The macrophage responses to pro- and anti-inflammatory mediators produced by various treatments were also measured (Table 5). For the cells without LPS stimulation, the model cells with LPS treatment only showed obvious increases in the secretion of the 4 inflammatory mediators, including NO, PGE2, TNF-α, and IL-6, which were nearly enhanced by 28-, 8-, 120-, and 448-fold, respectively. This fact indicated that LPS caused inflammation in the cells. However, if the cells were pre-treated with the CH, PCH, or PCH-Hyp at a dose level of 100 μg/mL, the cells after LPS stimulation showed a reduced inflammatory response because they were detected with less secretion in the 4 inflammatory mediators. Compared with the model cells, the cells treated by the CH, PCH, or PCH-Hyp totally caused about 14–32% decreases in NO production, 6–29% decreases in PGE2 production, 12–32% decreases in TNF-α production, and 22–47% decreases in IL-6 production, respectively. Therefore, the CH, PCH, and PCH-Hyp had anti-inflammatory capacity on the stimulated cells via efficiently inhibiting the secretion of the four inflammatory mediators. It also could be found that the modifiers with PCH, especially PCH-Hyp, had higher anti-inflammatory efficiency than CH, demonstrating that the plastein reaction (especially in the presence of Hyp) had a positive effect on the anti-inflammatory activity of the CH.

Meanwhile, LPS stimulation of the cells also resulted in higher production of anti-inflammatory mediators IL-10 and TGF-β1, which were increased from 14.6 and 23.7 pg/mL (control cells) to 254.9 and 214.3 pg/mL (model cells), respectively (Table 5). As expected, a treatment of the cells with the CH, PCH, and PCH-Hyp at 100 μg/mL induced more secretion of IL-10 (289.8–445.1 pg/mL) and TGF-β1 (219.3–332.0 pg/mL). The PCH-Hyp and CH led to the highest and lowest secretion levels of IL-10 and TGF-β1 in the cell, respectively, implying the plastein reaction (especially that with Hyp addition) efficiently promoted the anti-inflammatory capacity of the modifiers. Overall, all the results given in Table 5 consistently declared a fact; that is, the plastein reaction was beneficial to the anti-inflammatory activity of the CH in the LPS-stimulated cells, regarding its mediation on the secretion of these inflammatory and anti-inflammatory mediators in the cells.

### 3.4. Effect of the Modifiers on Genes and Proteins Involved in the NF-κB Signaling Pathway

To further reveal the potential anti-inflammatory effects of the CH and modifiers on the LPS-stimulated cells, the mRNA expression levels of eight genes (TLR4, iNOS, IL-6, TNF-α, IL-1β, COX-2, IL-10, and TGF-β1) and four micro genes (miR-181a, miR-30d, miR-155, and miR-148a) were examined. The results (Table 6) indicated that the model cells (exposed to LPS alone) had much higher mRNA expression levels of iNOS, IL-6, TNF-α, IL-1β, COX-2, TLR4, IL-10, and TGF-β1 (about 1.36–2.47-fold), compared with the control cells with set mRNA expression (1.00-fold) of these genes. Meanwhile, the model cells also had up-regulated miR-155 and miR-148a (1.55–1.87-fold) but down-regulated miR-181a and miR-30d (0.61–0.78-fold). The changes in the expression of these genes in the cells reflected the LPS-induced inflammation. However, the CH, PCH, or PCH-Hyp at 100 μg/mL showed an ability to down-regulate the expression of these pro-inflammatory genes, including iNOS, IL-6, TNF-α, IL-1β, COX-2, miR-155, and miR-148a, but up-regulate the anti-inflammatory genes, including IL-10, TGF-β1, miR-181a, and miR-30d, suggesting their anti-inflammatory effect on the LPS-stimulated cells. It was also seen that the PCH-Hyp and CH generally caused the highest and lowest regulation on expression levels of these genes, demonstrating that the plastein reaction (especially in the presence of Hyp) had a positive effect on the anti-inflammatory activity of the CH towards the LPS-stimulated cells.

**Figure 2:**
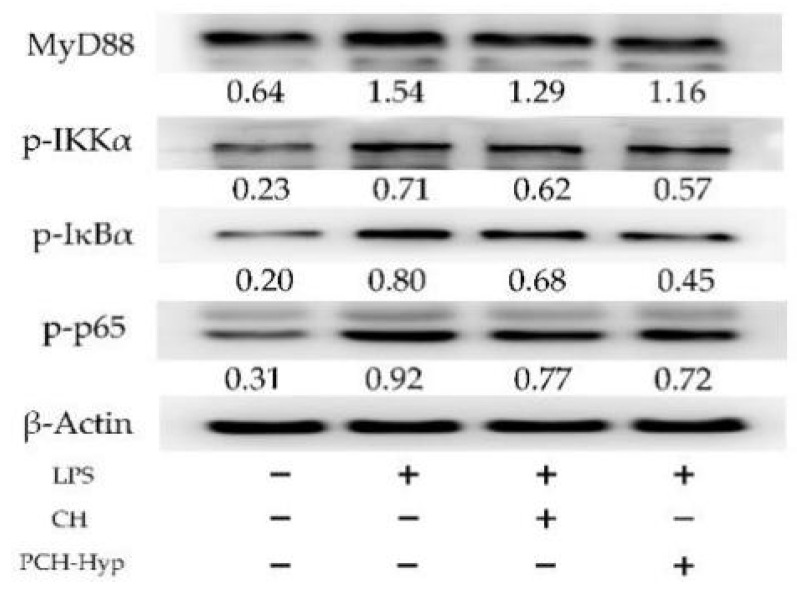
Effects of casein hydrolysates (CH) and the PCH-Hyp (the modifier of CH in the presence of Hyp) on the targeted protein expression involved in NF-κB signaling pathway.

The four key proteins, namely MyD88, p-IKKα, p-IκBα, and p-NF-κB p65, that are involved in the NF-κB signaling pathway, were detected using the Western blot assay. Compared with the control cells, the model cells in response to the LPS stimulation had up-regulated MyD88, p-IKKα, p-IκBα, and p-NF-κB p65 (0.71–1.54- versus 0.20–0.64-fold) (Figure 2). Thereby, LPS in the cells activated NF-κB signaling pathway and subsequently induced intracellular inflammation. However, cell exposure to the CH and PCH-Hyp (100 μg/mL dose) led to decreased MyD88 expression (1.16–1.29-fold) and reduced phosphorylation of IKKα, IκBα and p65 (0.45–0.77-fold). Furthermore, the PCH-Hyp was more active than the CH to regulate the expression of the four target proteins, indicating the performed plastein reaction gave CH a higher activity to regulate the expression of these target proteins.

### 3.5. Inhibition of the Modifiers on the NF-κB Signaling Pathway

**Figure 3:**
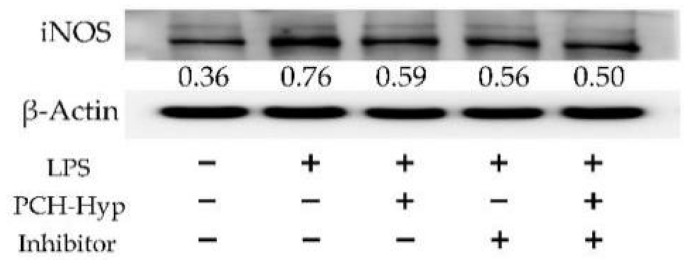
Inhibitory effects of the PCH-Hyp (the modifier of CH in the presence of Hyp) and specific NF-κB inhibitor BAY11-7082 on the expression of inducible nitric oxide synthase (iNOS).

To depict the anti-inflammatory function of the plastein modifier in cells, iNOS expression and p65 nuclear translocation of the NF-κB signaling pathway in the cells were evaluated. The Western blot results (Figure 3) confirmed that the model cells in response to LPS stimulation had much higher relative iNOS expression than the control cells (0.76 versus 0.36). However, the application of an inhibitor of NF-κB signaling pathway (i.e., BAY 11-7082) in the stimulated cells caused down-regulated relative iNOS expression (0.56). The PCH-Hyp at 100 μg/mL also had a similar effect on the stimulated cells by down-regulating relative iNOS expression to 0.59. Furthermore, there existed a synergetic effect between the PCH-Hyp and the inhibitor, because they led to much lower relative iNOS expression of 0.50. Therefore, it was evident that this modifier indeed had an anti-inflammatory effect on the stimulated cells via inhibition of NF-κB signaling pathway.

**Figure 4:**
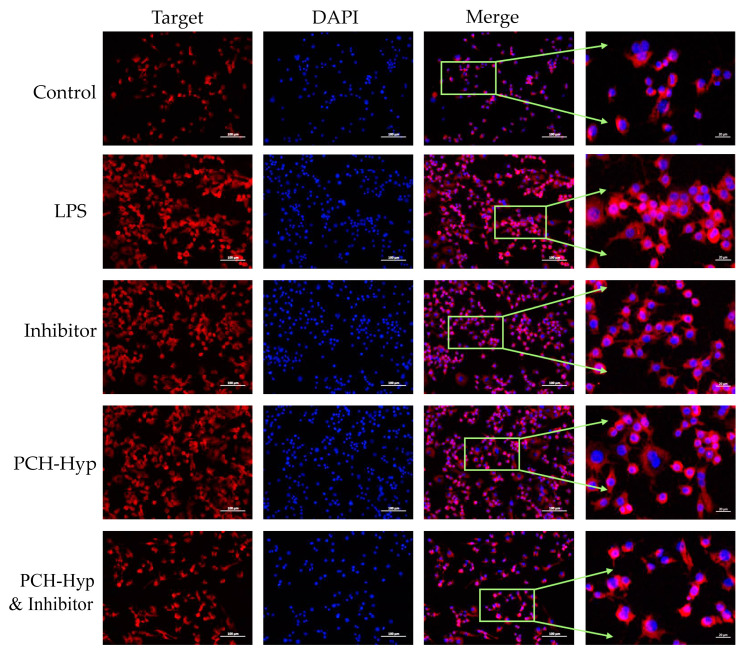
Nuclear translocation of p-p65 in the LPS-stimulated macrophages detected by an immuno-fluorescent staining. PCH-Hyp, the modifier of CH in the presence of Hyp; inhibitor, the specific NF-κB inhibitor BAY11-7082. The labeled bar is 100 μm for the original pictures but 20 μm for the partial pictures.

The same conclusion could be obtained when monitoring p65 nuclear translocation in the cells (Figure 4). In the control cells without LPS stimulation, the p65 protein was mainly expressed in the cytoplasm of the cells without obvious nuclear phenomenon. However, the nucleoplasmic portion of the model cells was fluorescently labeled, indicating that a large amount of p65 protein translocated into the nucleus (i.e., nuclear translocation of p65 protein). Compared with the model cells, the cells treated with the inhibitor (BAY 11-7082) or PCH-Hyp showed a blocked nuclear translocation of p65 protein, because the abnormal expression of p65 protein was clearly reduced. Moreover, there also existed a synergetic effect between the inhibitor and PCH-Hyp, because they together led to less nuclear translocation of p65 protein. The PCH-Hyp was thus regarded to possess a similar function as the inhibitor to inhibit nuclear translocation of the p65 protein. Hence, it was further confirmed that this modifier indeed had an anti-inflammatory effect on the stimulated cells by suppressing the activation of NF-κB signaling pathway.

## 4. Discussion

Protein hydrolysates or bioactive peptides generated from food proteins have been identified with various health-promoting properties, such as anti-oxidative, antibacterial, anti-cancer, anti-inflammatory, anti-hypertensive, immuno-regulatory, and other activities [33,34]. Furthermore, due to their potential neuroprotective and brain health effects, bioactive peptides have been suggested as a way to prevent cognitive decline and dementia [35]. More importantly, protein hydrolysates and bioactive peptides can be chemically modified to change their properties. For example, the porcine plasma protein hydrolysates modified by oxidized tannic acid or chlorogenic acid had higher antioxidation [36]. Moreover, it was also found that the oligochitosan-glycated casein hydrolysates by transglutaminase were more effective than the unglycated counterparts in the acrylamide-injured IEC-6 cells to protect critical barrier function [37]. The plastein reaction, due to its ability to induce peptide condensation and transpeptidation, is regarded to have the ability to alter the peptide sequences of protein hydrolysates, and shows the potential to modify bioactivities of protein hydrolysates. Subsequently, various protein hydrolysates subjected to plastein reaction were observed with enhanced activities to inhibit ACE, scavenge free radicals, or inhibit the growth of cancer cells [38,39,40]. Sharing a result similarity, this study also found that these modifiers had higher anti-inflammatory activities in the LPS-stimulated cells than the unmodified CH, evidencing that the plastein reaction might be a useful tool to improve peptide bioactivity again.

Several ingredients from natural foods also have been identified with anti-inflammatory function in the cells. The isolated heteropolysaccharide SHPS-1 from the fruiting bodies of *Phellinus baumii* has an anti-inflammatory effect on the LPS-stimulated RAW 264.7 cells through reducing the phosphorylation of STAT-1 and gene expression of iNOS and TNF-α [41]. More importantly, the expression of anti-inflammatory cytokine IL-10 and tissue repair gene CD 206 was also enhanced by SHPS-1, resulting in alleviated ulcerative colitis in mice [41]. It was found that the nuciferine from the lotus leaf could reduce the secretion of two pro-inflammatory mediators TNF-α and IL-1 in a dose-dependent manner to alleviate the LPS-induced inflammation in mouse mammary epithelial cells [36], while the flavonoids acacetin showed anti-inflammatory activity in the LPS-stimulated human periodontal ligament cells by decreasing the secretion of TNF-α, IL-6, and IL-1β [42]. Protein hydrolysates or bioactive peptides can exert a variety of anti-inflammatory activities on the cells. For instances, the buffalo casein peptides have been reported to have anti-inflammatory activity to the inflammatory spleen cells through reducing the secretion of INF-γ but increasing the levels of IL-10 and TGF-β [43], while the whey protein hydrolysates showed anti-inflammatory activities to the LPS-stimulated respiratory epithelial cells via affecting LPS binding to Toll-like receptor 4 and decreasing IL-8 secretion [44]. In addition, the soy protein hydrolysates evidently inhibited the iNOS/NO and COX-2/PGE2 pathways in the LPS-induced macrophages, mainly by reducing the generation of NO and PGE2 and the expression of iNOS and COX-2 [45]. Regarding the CH and the modifiers prepared in this study, they were both protein hydrolysates and thus reasonably had anti-inflammatory activities in the LPS-stimulated cells by regulating the secretion of these pro-inflammatory and anti-inflammatory mediators, as these mentioned studies indicated.

As usual, food components display their anti-inflammatory capacity in the cells via mediating several important signaling pathways, from a biological point of view. For example, the polysaccharides have been elucidated to inhibit cellular production of pro-inflammatory mediators through inhibition of NF-κB signaling pathway and reduction in p65 nuclear translocation [46,47]; for example, the garlic extract could reduce the production of NO in the LPS-stimulated RAW264.7 macrophages by inhibiting NF-κB nuclear translocation and HO-1 activation [48]. In addition, the fishbone protein hydrolysates showed an anti-inflammatory effect on the RAW264.7 macrophages by down-regulating the phosphorylation levels of JNK and NF-κB, resulting in a block in JNK/NF-κB signaling pathways [21], while lactoferrin could combat against the LPS-induced inflammation in human nasal epithelial cells via inhibition on MEK1/2-MAPK signaling pathway [49]. Overall, the TLR4/MyD88/NF-κB signaling pathway has attracted widespread attention in the scientific community due to their implant-related innate immune recognition and immuno-modulation [50,51]. In this study, it was found that LPS activated NF-κB signaling pathway and induced inflammation, as the previous study also found [52]. Meanwhile, in response to cell exposure of the prepared CH and modifiers, the cells showed regulated expression in these assessed proteins involved in the NF-κB signaling pathway, together with an inhibited nuclear translocation of p65 protein. Therefore, the CH and modifiers were regarded to alleviate the LPS-induced inflammation in the cells by suppressing the NF-κB signaling pathway activation and nuclear p65 translocation. Furthermore, the results using the inhibitor of the NF-κB signaling pathway provided extra evidence in this study to reveal the mediation of the CH and modifiers on NF-κB signaling pathway. Sharing a conclusion consistent with this study, it was reported that casein hydrolysate could inhibit the NF-κB signaling pathway activation and nuclear p65 translocation [53,54], or casein glycomacropeptides, could suppress NF-κB translocation in LPS-stimulated macrophages [55].

In recent years, growing attention has been paid to the potential nutrition and health functions of various food components. The plastein reaction has been employed for its potential to enhance the bioactivity of protein hydrolysates or bioactive peptides. However, it is also major challenge for us to reveal the potential structure–activity relationship of the plastein products. To be more specific, the reason that the modifiers prepared with exogenous amino acids (especially Hyp) had much higher activity towards the LPS-stimulated cells was not clarified in this study. In addition, whether or not the digested products have anti-inflammatory effects is still worthy of further investigation. Thus, a future investigation for this unsolved issue is necessary for us.

## 5. Conclusions

The results of the present study highlighted that the plastein reaction of the CH in the absence or presence of exogenous amino acids (especially Hyp) endowed the resultant modifiers with a higher potential to alleviate the LPS-induced inflammation in RAW264.7 macrophages. Compared with the unmodified CH or chemical mixtures of CH and the exogenous amino acids, the modifiers possessed a higher ability to promote macrophage growth and secretion of two anti-inflammatory mediators, and were more active to alleviate the LPS-promoted macrophage phagocytosis, LDH release, ROS formation, and the secretion of four inflammatory mediators. Moreover, it was also revealed from a biological point of view that the modifiers had a capacity to regulate the mRNA/miRNA/protein expression involved in the NF-κB signaling pathway, or to block the LPS-mediated nuclear translocation of p65 protein in the LPS-stimulated cells, as the NF-κB inhibitor BAY 11-7082 did. Both the plastein reaction and incorporation of exogenous amino acids (especially Hyp) contributed to the enhanced anti-inflammatory activity of the modifiers to attenuate the LPS-induced cell inflammation via suppressing NF-κB activation. In conclusion, the plastein reaction might be a promising way to induce bioactivity changes for CH including the target anti-inflammatory activity, but whether other hydrolysates subjected to this reaction might obtain bioactivity alteration should be further investigated.

## Figures and Tables

**Table 1:** The sequences of the primers used in RT-qPCR assays.

Genes	Primer Sequences (5′-3′)	Lengths of Output (bp)
iNOS	Forward: 5′-ACT CAG CCA AGC CCT CAC CTA C-3′ Reverse: 5′-TCC AAT CTC TGC CTA TCC GTC TCG-3′	111
IL-6	Forward: 5′-AGA CAG CCA CCA CAC TGG AGA TAG-3′ Reverse: 5′-CCT GCC TCC TGT TGA TGT GAA GTC-3′	149
TNF-α	Forward: 5′-GCC TCT TCT CAT TCC TGC TTG TGG-3′ Reverse: 5′-GTG GTT TGT GAG TGT GAG GGT CTG-3′	149
IL-1β	Forward: 5′-TCG CAG CAG CAC ATC AAC AAG AG-3′ Reverse: 5′-AGG TCC ACG GGA AAG ACA CAG G-3′	97
COX-2	Forward: 5′-GGT GCC TGG TCT GAT GAT GTA TGC-3′ Reverse: 5′-GGA TGC TCC TGC TTG AGT ATG TCG-3′	81
IL-10	Forward: 5′-TCCCTGGGTGAGAAGCTGAAGAC-3′ Reverse: 5′-CACCTGCTCCACTGCCTTGC-3′	96
TGF-β1	Forward: 5′-ACCGCAACAACGCCATCTATGAG-3′ Reverse: 5′-GGCACTGCTTCCCGAATGTCTG-3′	91
TLR4	Forward: 5′-CCGCTTTCACCTCTGCCTTCAC-3′ Reverse: 5′-ACCACAATAACCTTCCGGCTCTTG-3′	105
β-actin	Forward: 5′-CGCAAAGACCTGTATGCCAAT-3′ Reverse: 5′-GGGCTGTGATCTCCTTCTGC-3′	174
miR-181a	Forward: 5′-CGAACATTCAACGCTGTCG-3′ Reverse: 5′-AGTGCAGGGTCCGAGGTATT-3′	60
miR-30d	Forward: 5′-GCGTGTAAACATCCCCGAC-3′ Reverse: 5′-AGTGCAGGGTCCGAGGTATT-3′	60
miR-155	Forward: 5′-GCGCGTTAATGCTAATTGTGAT-3′ Reverse: 5′-AGTGCAGGGTCCGAGGTATT-3′	60
miR-148a	Forward: 5′-GCGCGTCAGTGCACTACAGAA-3′ Reverse: 5′-AGTGCAGGGTCCGAGGTATT-3′	60
U6	Forward: 5′-AGGTATTCGCACTGGATACGAC-3′ Reverse: 5′-AGTGCAGGGTCCGAGGTATT-3′	60

**Table 2:** The classification and free −NH_2_ contents of the prepared samples used in this study.

Sample	Classification	−NH_2_ Contents (mmol/g Protein)
CH	Casein hydrolysates	1.160 ± 0.002 ^f^
PCH	The modifier of CH	0.935 ± 0.002 ^g^
PCH-Gly	The modifier of CH and Gly	1.433 ± 0.003 ^e^
PCH-Pro	The modifier of CH and Pro	1.451 ± 0.004 ^d^
PCH-Hyp	The modifier of CH and Hyp	1.456 ± 0.003 ^d^
CH-Gly	The mixture of CH and Gly	1.792 ± 0.006 ^c^
CH-Pro	The mixture of CH and Pro	1.807 ± 0.006 ^b^
CH-Hyp	The mixture of CH and Hyp	1.820 ± 0.005 ^a^

Different lowercase letters as superscripts after the mean values indicate that one-way ANOVA of the mean values is significantly different (*p* < 0.05).

**Table 3:** Effects of casein hydrolysate (CH), its plastein modifiers and mixtures at three assessed dose levels on the growth proliferation of the macrophages reflected by the measured cell viability (%).

Sample	Cell Viability at Different Dose Levels (μg/mL)		
	25	50	100
CH	102.5 ± 2.2 ^Ae^	110.2 ± 3.7 ^Bd^	111.7 ± 3.5 ^Bd^
PCH	111.8 ± 4.6 ^Aabcd^	115.2 ± 4.9 ^Abcd^	119.1 ± 1.8 ^Abc^
PCH-Gly	114.3 ± 6.3 ^Aab^	121.3 ± 5.5 ^Aab^	124.4 ± 2.6 ^Ab^
PCH-Pro	113.7 ± 3.9 ^Babc^	119.6 ± 4.2 ^ABabc^	122.9 ± 3.5 ^Ab^
PCH-Hyp	115.4 ± 5.6 ^Ba^	125.9 ± 4.1 ^Aa^	129.7 ± 2.2 ^Aa^
CH-Gly	103.9 ± 2.5 ^Bde^	112.5 ± 5.5 ^Abcd^	113.3 ± 4.2 ^Ad^
CH-Pro	105.6 ± 3.3 ^Acd^	112.1 ± 6.0 ^Acd^	112.7 ± 1.8 ^Ad^
CH-Hyp	106.4 ± 4.9 ^Bbcde^	113.7 ± 2.8 ^ABbcd^	114.5 ± 3.3 ^Acd^

PCH—the modifier of CH without exogenous amino acids; PCH-Gly (PCH-Pro or PCH-Hyp)—the modifier of CH in the presence of Gly (Pro or Hyp); CH-Gly (CH-Pro or CH-Hyp)—the mixture of CH and Gly (Pro or Hyp). Different uppercase (or lowercase) letters as superscripts after the mean values in the same row (or column) indicate that one-way ANOVA of the mean values is significantly different (*p* < 0.05).

**Table 4:** Effects of casein hydrolysate (CH) and its modifiers and mixtures at three assessed dose levels on the phagocytic index (PI) of the macrophages.

Sample	Dose Levels (μg/mL)		
	25	50	100
CH	2.01 ± 0.04 ^Aa^	1.96 ± 0.03 ^Aa^	1.95 ± 0.05 ^Aa^
PCH	1.77 ± 0.05 ^Ac^	1.74 ± 0.03 ^Ac^	1.72 ± 0.03 ^Ac^
PCH-Gly	1.73 ± 0.04 ^Ac^	1.61 ± 0.04 ^Bd^	1.61 ± 0.045 ^Bd^
PCH-Pro	1.75 ± 0.06 ^Ac^	1.71 ± 0.05 ^Ac^	1.70 ± 0.06 ^Ac^
PCH-Hyp	1.71 ± 0.04 ^Ac^	1.55 ± 0.02 ^Bd^	1.52 ± 0.04 ^Be^
CH-Gly	1.94 ± 0.05 ^Aab^	1.90 ± 0.05 ^Aab^	1.87 ± 0.03 ^Aab^
CH-Pro	1.93 ± 0.06 ^Aab^	1.92 ± 0.04 ^Acd^	1.90 ± 0.04 ^Aa^
CH-Hyp	1.87 ± 0.04 ^Ab^	1.86 ± 0.04 ^Ab^	1.81 ± 0.06 ^Ab^

PCH—the modifier of CH without exogenous amino acids; PCH-Gly (PCH-Pro or PCH-Hyp)—the modifier of CH in the presence of Gly (Pro or Hyp); CH-Gly (CH-Pro or CH-Hyp)—the mixture of CH and Gly (Pro or Hyp). Different uppercase (or lowercase) letters as superscripts after the mean values in the same row (or column) indicate that one-way ANOVA of the mean values is significantly different (*p* < 0.05).

**Table 5:** The secretion levels (μmol/L for NO and ng/L for others) of the inflammatory and anti-inflammatory mediators in the macrophages treated with or without the LPS, casein hydrolysates, or modifiers.

Mediator	The Cells with Different Treatments				
	Control	LPS	CH	PCH	PCH-Hyp
NO	1.6 ± 0.3 ^e^	45.7 ± 1.0 ^a^	39.3 ± 0.6 ^b^	36.9 ± 0.8 ^c^	31.3 ± 0.9 ^d^
PGE2	21.8 ± 2.9 ^e^	169.2 ± 3.3 ^a^	158.4 ± 3.2 ^b^	148.2 ± 2.2 ^c^	119.3 ± 2.8 ^d^
TNF-α	32.8 ± 1.2 ^e^	3945.8 ± 60.8 ^a^	3490.2 ± 71.3 ^b^	3266.7 ± 65.6 ^c^	2673.4 ± 49.3 ^d^
IL-6	1.5 ± 0.1 ^e^	673.0 ± 14.8 ^a^	527.4 ± 15.3 ^b^	456.7 ± 15.9 ^c^	354.5 ± 9.2 ^d^
IL-10	14.6 ± 2.1 ^e^	254.9 ± 9.4 ^d^	289.8 ± 6.2 ^c^	322.5 ± 8.0 ^b^	445.1 ± 8.9 ^a^
TGF-β1	23.7 ± 4.5 ^d^	214.3 ± 5.2 ^c^	219.3 ± 4.8 ^c^	246.5 ± 6.7 ^b^	332.0 ± 8.1 ^a^

CH—casein hydrolysates; PCH—the modifier of CH without exogenous amino acids; PCH-Hyp—the modifier of CH in the presence of Hyp. Different lowercase letters as superscripts after the mean values in the same row indicate that one-way ANOVA of the mean values is significantly different (*p* < 0.05).

**Table 6:** Relative mRNA and miRNA expression of the macrophages with different treatments.

Gene	The Cells with Different Treatments				
	Control	LPS	CH	PCH	PCH-Hyp
iNOS	1.00	2.41 ± 0.04	2.29 ± 0.03	1.93 ± 0.16	1.71 ± 0.12
IL-6	1.00	2.47 ± 0.05	2.28 ± 0.04	1.92 ± 0.28	1.84 ± 0.09
TNF-α	1.00	1.89 ± 0.08	1.81 ± 0.09	1.7 3± 0.14	1.68 ± 0.03
IL-1β	1.00	2.25 ± 0.13	2.19 ± 0.11	2.03 ± 0.07	1.80 ± 0.08
COX-2	1.00	2.14 ± 0.03	1.97 ± 0.05	1.73 ± 0.04	1.67 ± 0.05
TLR4	1.00	1.91 ± 0.02	1.86 ± 0.15	1.79 ± 0.13	1.73 ± 0.07
IL-10	1.00	1.45 ± 0.02	1.53 ± 0.12	1.61 ± 0.19	1.68 ± 0.12
TGF-β1	1.00	1.36 ± 0.06	1.38 ± 0.07	1.47 ± 0.02	1.51 ± 0.11
miR-181a	1.00	0.78 ± 0.04	1.43 ± 0.08	1.51 ± 0.06	1.75 ± 0.06
miR-30d	1.00	0.61 ± 0.05	0.67 ± 0.04	0.74 ± 0.04	0.77 ± 0.09
miR-155	1.00	1.87 ± 0.06	1.80 ± 0.04	1.66 ± 0.04	1.59 ± 0.02
miR-148a	1.00	1.55 ± 0.19	1.51 ± 0.03	1.45 ± 0.07	1.37 ± 0.15

The relative expression levels of miR-181a, miR-30d, miR-155, and miR-148a were normalized to U6 snRNA, while those levels of other genes were normalized to β-actin.

## Data Availability

All data are contained within the article.
